# Fluorouracil-based neoadjuvant chemoradiotherapy with or without oxaliplatin for treatment of locally advanced rectal cancer: An updated systematic review and meta-analysis

**DOI:** 10.18632/oncotarget.9995

**Published:** 2016-06-14

**Authors:** Yong-Jing Yang, Ling Cao, Zhi-Wen Li, Ling Zhao, Hong-Fen Wu, Dan Yue, Jin-Lei Yang, Zhi-Rui Zhou, Shi-Xin Liu

**Affiliations:** ^1^ Department of Radiation Oncology, Cancer Hospital of Jilin Province, Changchun, 130012, People's Republic of China; ^2^ Department of Anesthesiology, The First Hospital Affiliated to Jilin University, Changchun, 130012, People's Republic of China; ^3^ Department of Radiation Oncology, Fudan University Shanghai Cancer Center, Shanghai, 200032, People's Republic of China

**Keywords:** rectal neoplasms, neoadjuvant chemoradiotherapy, oxaliplatin, meta-analysis

## Abstract

To measure the safety and efficacy of oxaliplatin (OX) application in neoadjuvant chemoradiotherapy (CRT) for locally advanced rectal cancer (LARC), EMBASE, PubMed, Cochrane Library, and Web of Science were used for a literature search. Cochrane's risk of bias tool of randomized controlled trials (RCTs) was used for quality evaluation. The statistical analyses were performed using RevMan 5.3. In addition, 95% confidence intervals (CIs) and pooled risk ratios (RRs) were calculated. Seven RCTs were included in our meta-analysis. After adding OX to fluoropyrimidine (FU), a marginal significant improvement in disease-free survival was noted compared with FU alone (RR = 0.89, 95% CI: 0.78–1.00; *P* = 0.05). Neoadjuvant CRT with OX significantly decreased the distant metastasis rate (RR = 0.79, 95% CI: 0.67–0.94, *P* = 0.007). However, no improvement in the local recurrence rate (RR = 0.86, 95% CI: 0.68–1.08; *P* = 0.19) was noted. In addition, neoadjuvant CRT with OX also significantly increased the pathologic complete response (RR = 1.24, 95% CI: 1.02–1.51; *P* = 0.03). Grade 3–4 acute toxicity and grade 3–4 diarrhea was considerably higher for OX/FU compared with FU alone. In conclusion, the use of OX on the basis of FU/capecitabine in preoperative CRT is feasible. LARC patients are likely to benefit from CRT regimens with OX.

## INTRODUCTION

Colorectal cancer ranks as the third highest malignant tumor and the third leading cause of cancer death in the United States [1]. In 2014, 71,830 men and 65,000 women were estimated to be diagnosed with colorectal cancer, and 26,270 men and 24,040 women died of locally advanced rectal cancer (LARC) [1]. Thus, this disease is a major threat to people's health [2].

At present, fluoropyrimidine (FU) (5-fluorouracil and capecitabine)-based preoperative chemoradiotherapy (CRT) represents the standard of care for the treatment of LARC. Nevertheless, FU-based preoperative CRT only provides a significant reduction in local recurrence and not the distant metastasis rate [2, 3]. To improve the efficacy in the treatment of LARC, the drug selection for neoadjuvant concurrent chemotherapy has become a research focus in recent years [2, 4]. Oxaliplatin (OX) is a third generation, platinum drug that has been proven to be an ideal radiosensitizer *in vitro* and *in vivo* experiments [5–9] and has been widely applied in adjuvant therapy for rectal carcinoma [10, 11]. Thus, an increasing number of investigators have focused on OX with the expectation that it will improve the efficacy of neoadjuvant chemotherapy in LARC. The safety and efficacy of OX in neoadjuvant chemoradiotherapy for LARC has been studied in a number of phase II and III clinical trials [12–22]. In these studies, high pathologic complete response (pCR) rates were obtained (some trials even reached up to 28%). In addition, adverse reactions significantly increased, but most of these were tolerable during CRT. The maximum tolerated weekly dose of OX plus FU/capecitabine was 60 or 50 mg/m^2^ [12, 14–20, 23].

A debate on whether the addition of OX as a neoadjuvant modality improves the clinical outcomes for LARC is ongoing [24]. To date, seven phase III randomized controlled trials investigated the effect of OX in FU/capecitabine-based neoadjuvant therapy for LARC: STAR-01 in Italy [17], ACCORD12/0405 in France [18, 25], NSABP R-04 in the USA [16, 19], CAO/ARO/AIO-04 in Germany [4, 20], JIAO 2015 [21] and FOWARC study [22] in China, and PETACC-6 (NCT00766155) in Europe [26, 27]. However, these studies did not reach a consistent conclusion, especially regarding whether the addition of oxaliplatin improves disease-free survival (DFS). In 2013, a meta-analysis assessed this issue [28]. However, because most included trials did not obtain long-term survival results, the meta-analysis only summarized and analyzed the indicators of short-term effects, such as pathologic complete response (pCR). In addition, no long-term follow-up outcomes, such as DFS, were reported. To further ascertain the long-term survival effect and the safety of oxaliplatin application in neoadjuvant chemoradiotherapy for LARC, we performed an updated systematic review and meta-analysis.

## RESULTS

### Included study

**Figure 1:**
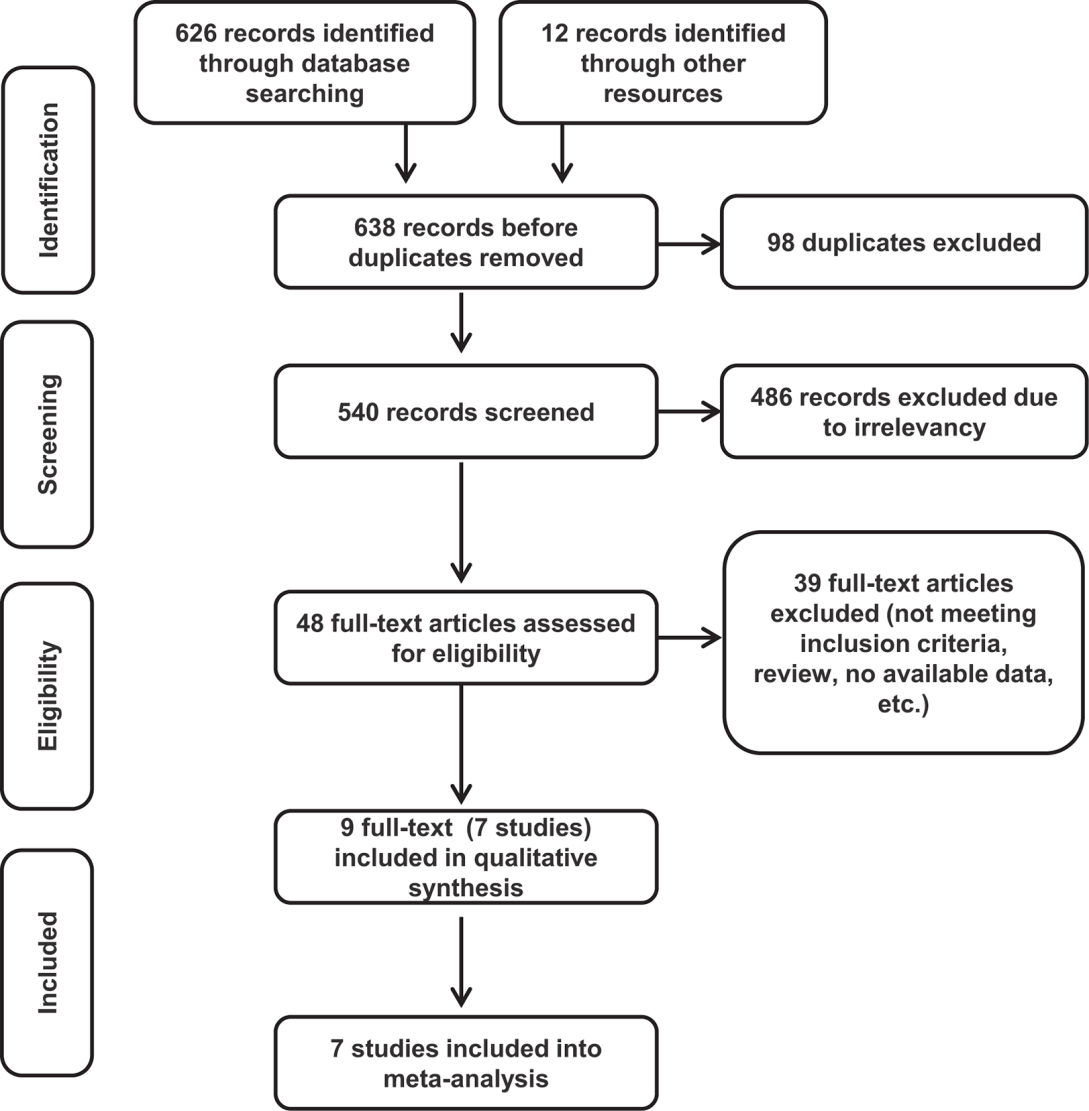
Flow chart of the study selection process

Using the search strategy as shown in appendix, we retrieved 638 records, among which 98 records were excluded as duplicates using the “find duplicates” feature of EndNote X7. The remaining 540 records were reviewed by titles and abstracts, among which 486 were excluded because they were irrelevant and did not meet the inclusion criteria. To further determine eligibility, 46 full-text articles and 2 meeting abstracts were acquired. We then excluded an additional 39 records. Specifically, 31 records were removed because patients or interventions did not meet the inclusion criteria. Six articles were removed because they are review articles. The remaining 2 articles were removed because data were not available. The meta-analysis included 7 trials, with a total of 5415 patients: STAR-01 trial [17], ACCORD12/0405 trial [18, 25], NSABP R-04 trial [16, 19], CAO/ARO/AIO-04 trial [4, 20], JIAO 2015 [21], FOWARC study [22] and PETACC-6 trial [26, 27]. Figure 1 shows the literature screening process, and Table 1 reveals the characteristics of the included studies.

**Table 1:** Baseline characteristics of included trials

Study	Sample Size		Chemotherapy regimens		Radiation	Adjuvant Chemotherapy	Follow-up time Median
	Treatment Group	Control Group	Treatment Group	Control Group			
STAR-1, 2011 [17]	368	379	5-FU + OX:	5-FU:225 mg/m2/d	50.4Gy/28f	Fluorouracil-based	/
			60 mg/m2/w×6				
ACCORD, 2012	299	299	CAPE + OX:	CAPE:	CAPE:45Gy/25f;	Fluorouracil-based	36.8 m
[18, 25]			50 mg/m2/w × 5	800 mg/m2,bid,5d/w	CAP/OX:50Gy/25f		
NSABP R-04, 2014	5-FU + OX:329;	5-FU:477;	5-FU/CAPE + OX:	5-FU:225 mg/m2/d,5d/w or	45Gy/25f + Boost:	Not specified	/
[16, 19]	CAPE/OX:330	CAPE:472	50 mg/m2/w × 5	CAPE:825 mg/m2,bid,5d/w	(5.4–10.8Gy)		
CAO/ARO/	613	623	5-FU: 250 mg/m^2^	5-FU:1000 mg/m2,	50.4Gy/28f	Control: Fluorouracil intravenous bolus	50 m
AIO-04, 2015 [4, 20]			d1-14 and 22–35	d1-5,d29-33		500 mg/m2, day 1–5 and 29, 4 cycles;	
			+OX: 50 mg/m2,			Treatment: OX+LV+FU, day 1	
			d1,8,22,29			and 15, 8 cycles	
PETACC-6, 2014	526	543	CAPE+OX:	CAPE:	45Gy/25f ± Boost:	Control: CAPE 1000 mg/m2,	31 m
[26, 27]			50 mg/m2/w×5	825 mg/m2,bid,5d/w	(5.4Gy)	bid,d1-15, Q3w; Treatment:	
						CAPE+OX: 100 mg/m2/d, Q3w	
JIAO et al, 2015	103	103	CAPE+OX:	CAPE:800 mg/m2, bid.,	50.0Gy/25f	All patients: 6−8 cycles FOLFOX	48.7 m
[21]			60 mg/m2,	d1-14,d22-35			
			d1,8,22,29				
FOWARC, 2015 [22]	165	165	mFOLFOX6	5-FU	46–50.4Gy	Control: De Gramont, 7cycles	/
					5f/week x 5-6w	Treatment: mFOLFOX6, 7cycles	

CAPE: capecitabine; 5-FU, flourouracil; NSABP, National Surgical Adjuvant Breast and Bowel Project; bid: twice daily.

OX, oxaliplatin; LARCs: Locally Advanced Rectal Cancer; d:day, w:week, m:month.mFOLFOX6:5-FU,tetrahydrofolic acid, oxaliplatin.

### Methodological quality of included studies

**Figure 2:**
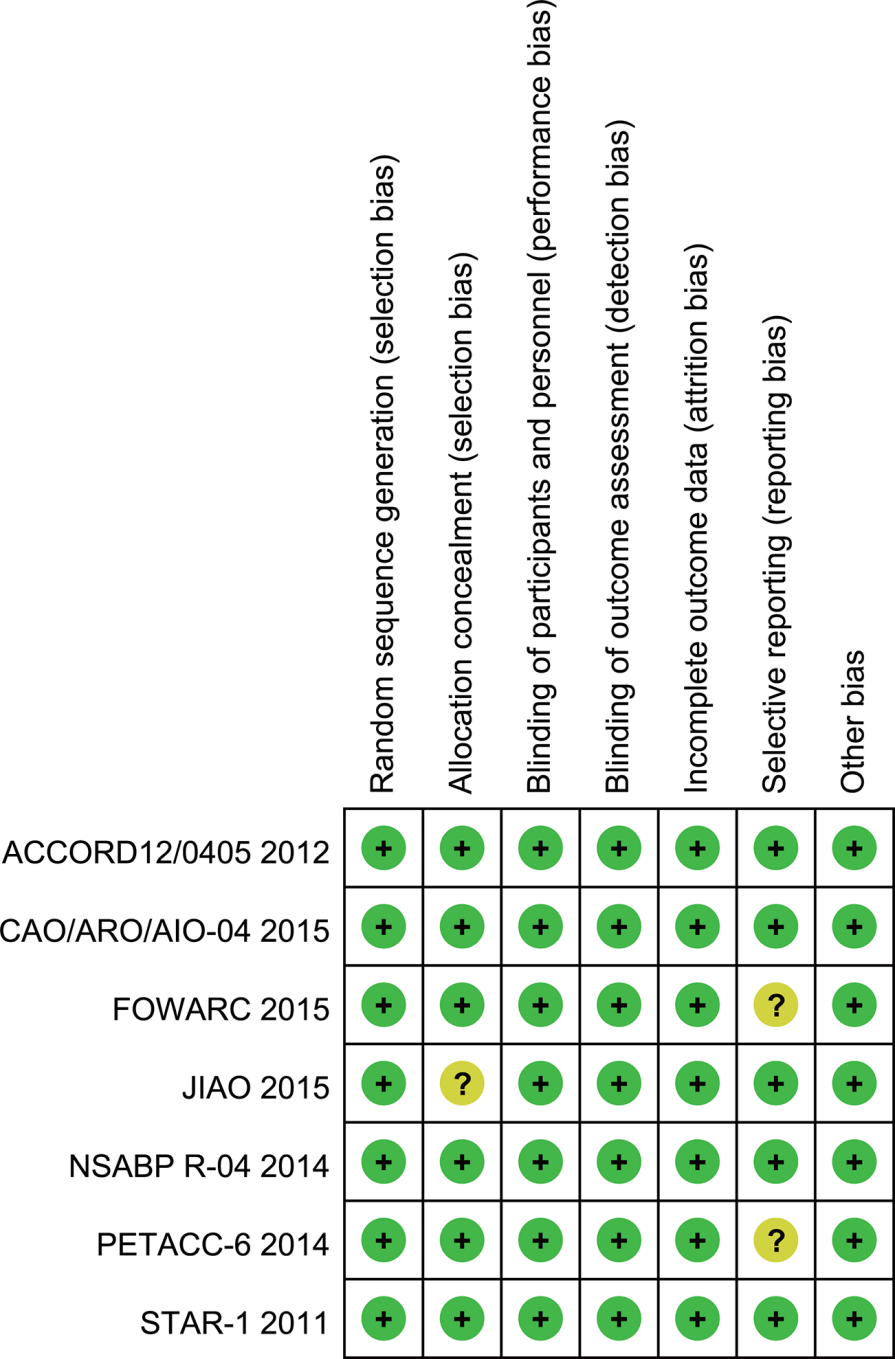
Risk of bias summary A review of the authors' judgments about each risk of bias item for each included study.

This systematic review included 7 RCTs. Each included RCT was subject to quality assessment in accordance with the Handbook of Cochrane for Systematic Reviews of Interventions [29]. The baseline characteristics of patients were reported in all RCTs. All studies mentioned “random”. All studies reported an adequate randomized sequence generation. Six studies reported methods of allocation concealment. All trials described the reasons of incomplete outcome data. Two abstracts did not report long-term follow-up outcomes according to their protocol currently; thus, a selective report bias may be present [22, 26]. Figure 2 presents the quality of all evaluated trials that were included.

### Disease-free survival

**Figure 3:**
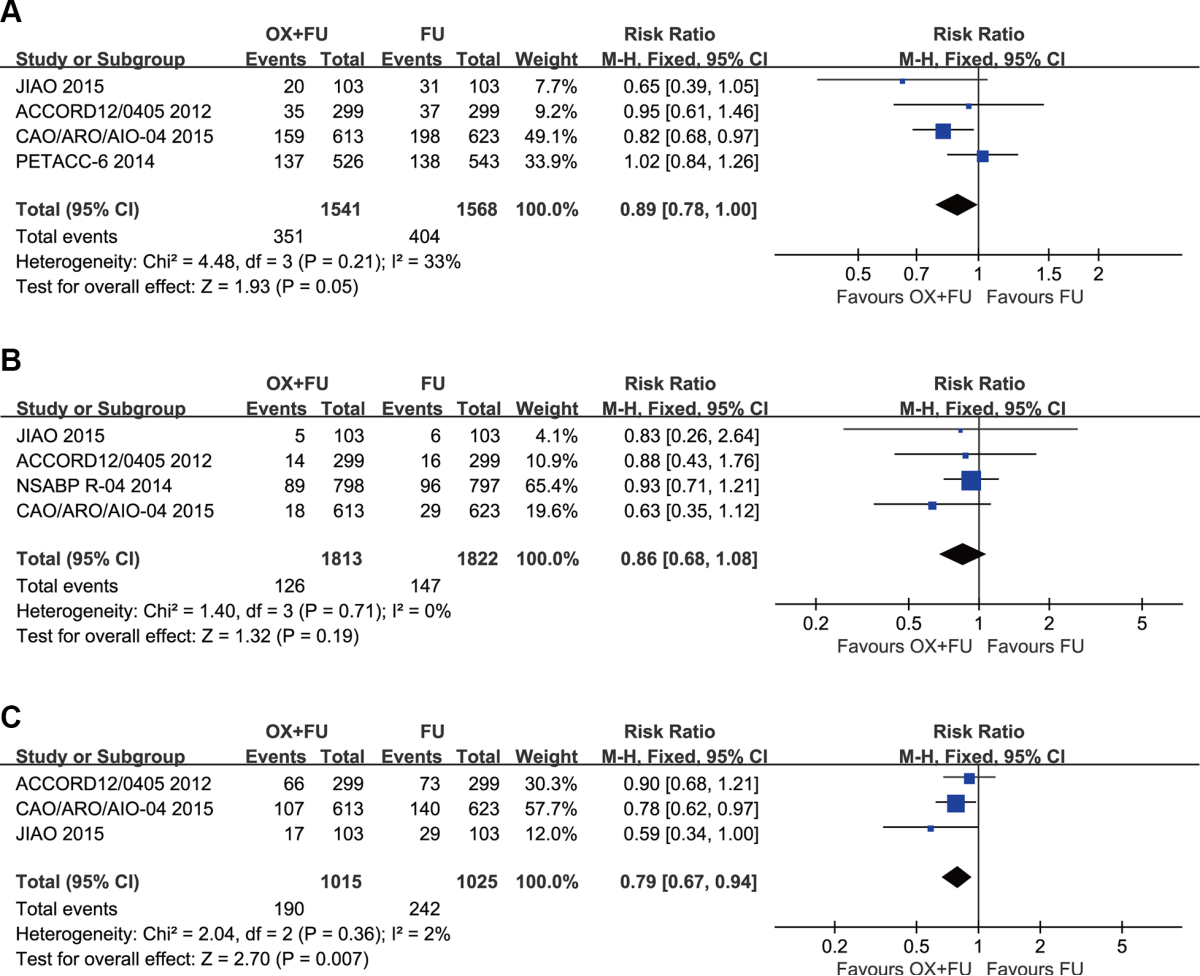
(**A**) Forest plot of risk ratio for DFS; (**B**) Forest plot of risk ratio for LRR; (**C**) Forest plot of risk ratio for DMR.

Disease-free survival results were reported in 4 studies, and a total of 3109 patients with rectal cancer were included in our study. The 3-year DFS rates of these studies ranged between 72.7% and 75.9% for the OX/FU programs. For the FU only regimens, the range was 67.9% to 74.5%. A marginal significant difference between patients treated with OX/FU programs and FU only programs (RR = 0.89, 95% CI: 0.78–1.00; *P* = 0.05) was noted in the meta-analysis for DFS. Based on heterogeneity (Chi² = 4.48, *P* = 0.21; *I*² = 33%), a fixed-effect model was adopted (Figure 3A).

### Local recurrence rate

Local recurrence rates were reported in 3 studies with a total of 2399 patients with rectal cancer; all three studies were included in this study. The 3-year LR rates ranged from 2.9% to 11.2% for the OX/FU programs. Regarding the FU only programs, the range was from 4.6% to 12.1%. No significant difference was noted between the OX/FU and FU alone groups (RR = 0.86, 95% CI: 0.68–1.08; *P* = 0.19) in the meta-analysis for local recurrence rated. Based on heterogeneity, (Chi² = 1.40, *P* = 0.71; *I*² = 0%), the fixed-effect model was performed (Figure 3B).

### Distant metastasis rate

Three trials reported the distant metastasis rate. A total of 2040 patients were included in the meta-analysis. OX/FU significantly decreased the distant metastasis rate compared with FU alone (RR=0.79, 95% CI: 0.67–0.94, *P* = 0.007). Heterogeneity was not detected (Chi² = 2.04, *P* = 0.36; *I*² = 2%), so the fixed-effect model was applicable (Figure 3C).

### pCR rate

**Figure 4:**
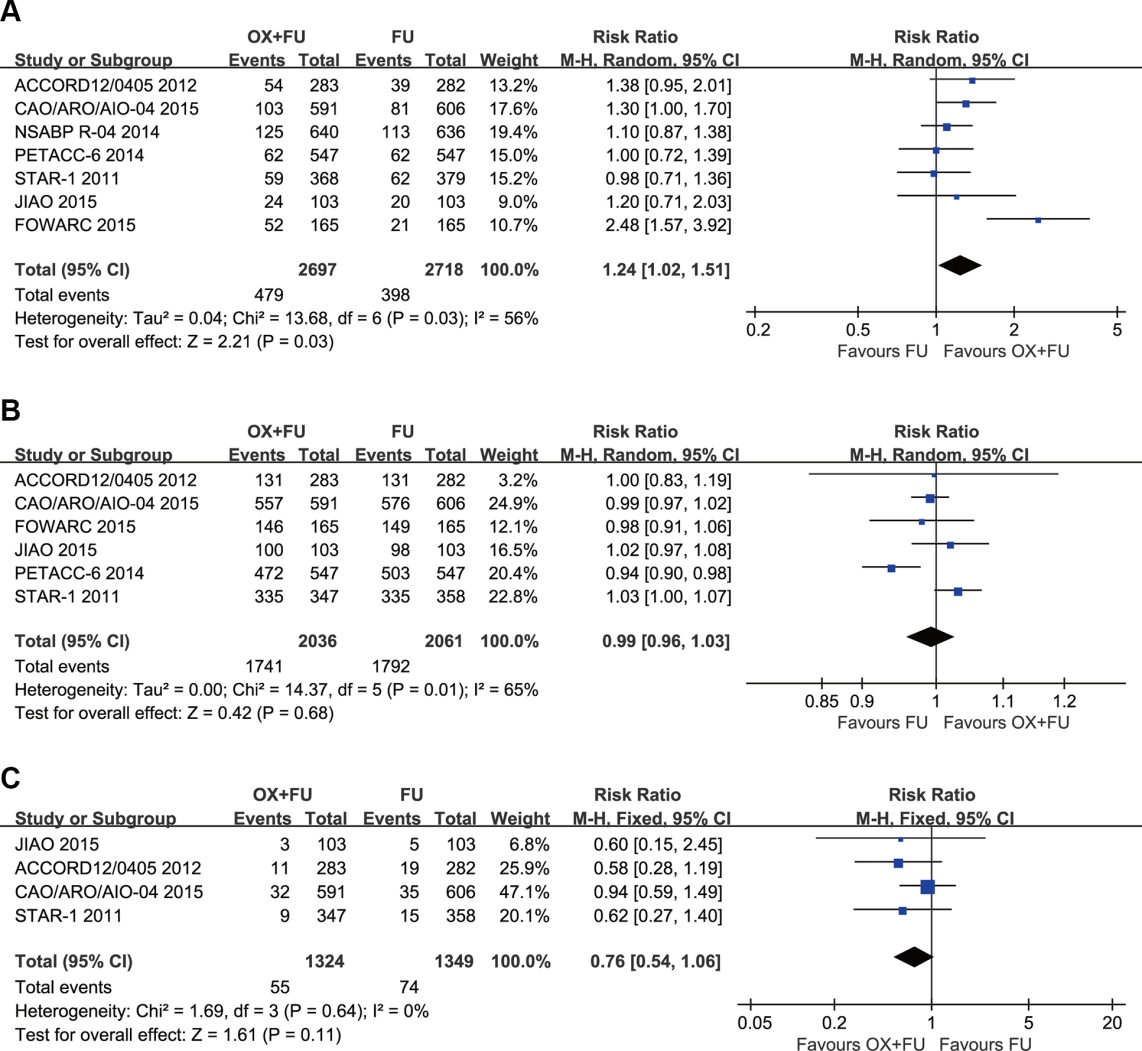
(**A**) Forest plot of risk ratio for pCR; (**B**) Forest plot of risk ratio for R0 resection; (**C**) Forest plot of risk ratio for CRM (+).

All included trials reported the pCR rate. A total of 5415 patients were included in the meta-analysis. The range of pCR rates was 11.3% to 19.5% for the OX/FU regimens and from 11.3% to 17.8% for the FU only programs. Significant heterogeneity was calculated (Tau² = 0.04; Chi² = 13.68, *P* = 0.03; *I*² = 56%). Thus, a pooled analysis was performed using the random-effect model. Our meta-analysis revealed that the OX/FU significantly increased the pCR rate compared with the FU only arms (RR =1.24, 95% CI: 1.02–1.51; *P* = 0.03) (Figure 4A).

### R0 resection rate

The R0 resection rate was reported in 6 trials with a total of 4097 patients incorporated in this meta-analysis. Significant heterogeneity was detected (Tau² = 0.00; Chi² = 14.37, *P* = 0.01*; I*² = 65%), so a random-effect model was adopted. Our meta-analysis revealed no significant difference in R0 resection rates between the two groups (RR=0.99, 95% CI: 0.96–1.03; *P* = 0.68) (Figure 4B).

### CRM status

Four studies reported circumferential rectal margin (CRM) status; the meta-analysis included a total of 2673 patients. The CRM (+) rate ranged from 4.0% to 7.7% for the OX/FU groups and from 6.0% to 12.7% for the FU only groups. The pooled estimate using the fixed-effect model indicated no significant difference between the two programs (RR =0.76, 95% CI: 0.54–1.06; *P* = 0.11) (Figure 4C).

### Grade 3–4 acute toxicity

**Figure 5:**
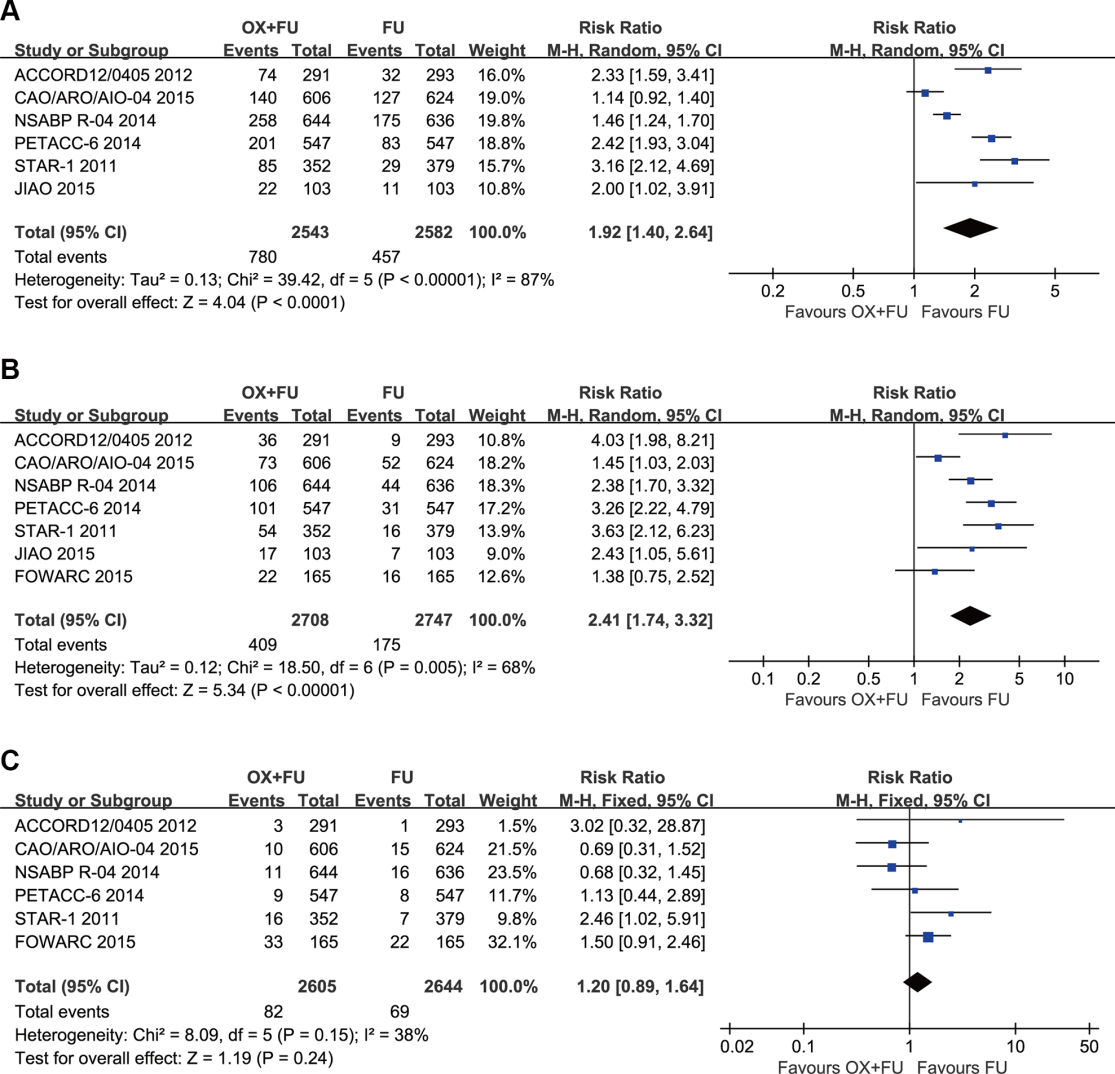
(**A**) Forest plot of risk ratio for grade 3–4 acute toxicity; (**B**) Forest plot of risk ratio for Grade 3–4 diarrhea; (**C**) Forest plot of risk ratio for grade 3–4 radiation dermatitis.

Grade 3–4 acute toxicity was reported in 6 trials; the meta-analysis included a total of 5125 patients with LARC. The rates of grade 3–4 acute toxicity ranged from 23.0% to 40.1% for the OX/FU arms and from 8% to 29.8% for the FU only arms. As a result of substantial heterogeneity (Tau² = 0.13; Chi² = 39.42, *P* < 0.00001; *I*² = 87%), we adopted a random-effect model. Our meta-analysis results indicated that OX/FU regimens significantly increased 3–4 grade acute toxicities compared with FU only regimens (RR = 1.92, 95% CI: 1.40–2.64;*P* < 0.0001) (Figure 5A).

### Grade 3–4 diarrhea

Seven trials described grade 3–4 diarrhea; the meta-analysis included total of 5455 patients with LARC. The rates of the grade 3–4 diarrhea ranged between 12.0% and 18.4% for the OX/FU arms and between 3.2% and 8.0% for the FU only arms. As a result of significant heterogeneity (Tau² = 0.12; Chi² = 18.50, *P* = 0.005; *I*² = 68%), a random-effect model was adopted. Our meta-analysis results indicated that OX/FU regimens significantly increased grade 3–4 diarrhea compared with FU only regimens (RR =2.41, 95% CI: 1.74–3.32;*P* < 0.00001). (Figure 5B).

### Grade 3–4 radiation dermatitis

Six studies reported grade 3–4 radiation dermatitis. The rates of grade 3–4 radiation dermatitis ranged between 1.4% and 5% for the OX/FU programs and from 0.4% to 3.2% for the FU alone programs. Pooled estimate adopted the fixed-effect model, which included 5249 LARC patients who were assessed for grade 3–4 radiation dermatitis. The pooled results indicated no significant difference between the two groups (RR = 1.20, 95% CI: 0.89–1.64; *P* = 0.24). Heterogeneity: Chi² = 8.09, *P* = 0.15; *I*² = 38% (Figure 5C).

### Grade 3–4 hematological toxicity

**Figure 6:**
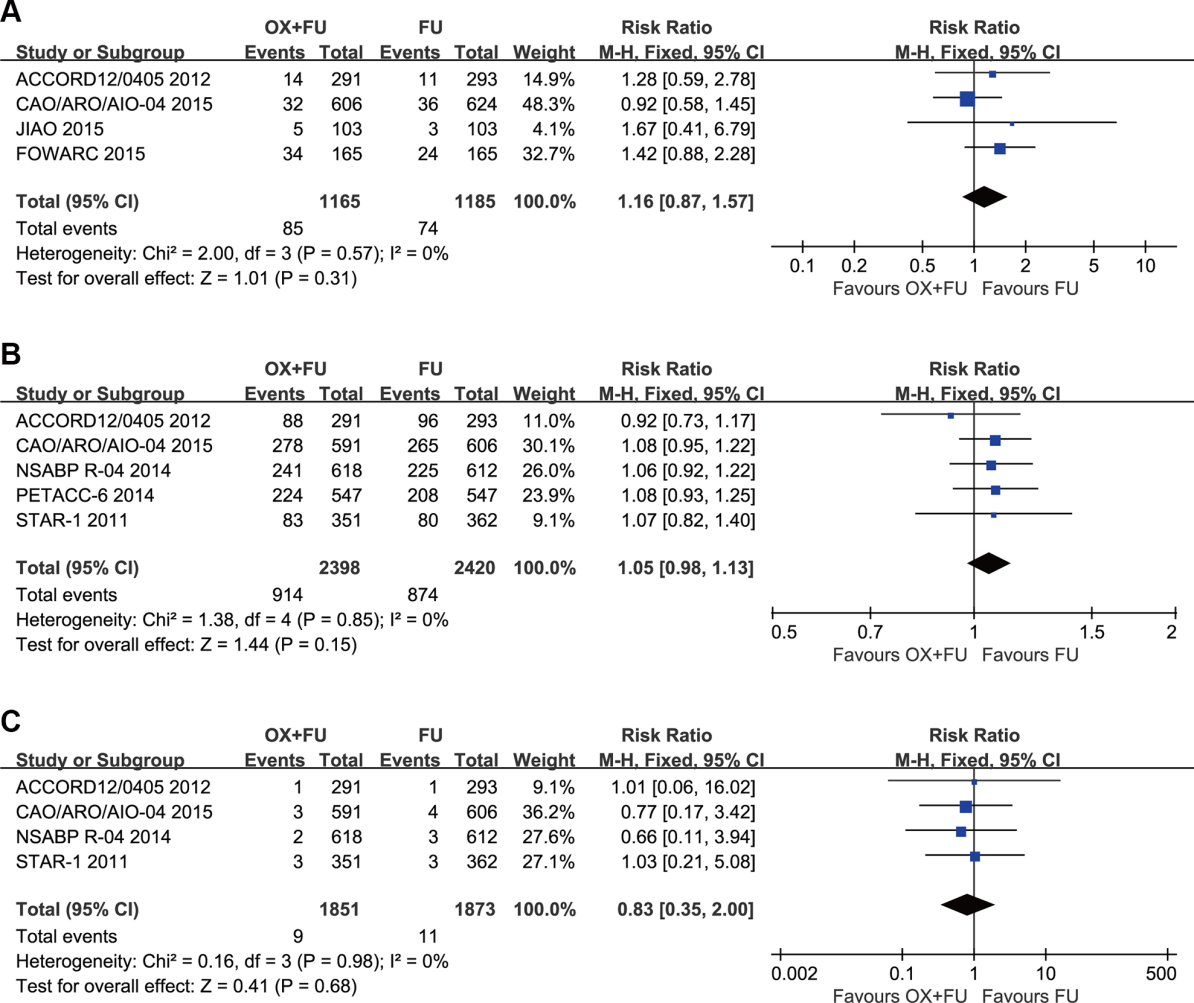
(**A**) Forest plot of risk ratio for grade 3–4 hematologic toxicity; (**B**) Forest plot of risk ratio for postoperative complications; (**C**) Forest plot of risk ratio for death within 60 days.

Grade 3–4 hematological toxicities were reported in 4 studies and their rates ranged between 4.8% and 5% for the OX/FU programs and from 3.7% to 6% for the FU alone programs. Pooled estimate adopted the fixed-effect model. A total of 2350 patients assessed for grade 3–4 hematological events. The results of our study indicated no significant difference between the two arms (RR = 1.16, 95% CI: 0.87–1.57; *P* = 0.31). Heterogeneity: Chi² = 2.00, *P* = 0.57; *I*² = 0%. (Figure 6A).

### Postoperative complication rate

Postoperative complications were reported in five studies. Postoperative complication rates ranged between 24% and 47% for the OX/FU programs and from 22% to 44% for the FU alone programs. A fixed-effect model was used for pooled analyses, which included 4818 patients assessed for postoperative complications. No significant difference was found between the two programs (RR = 1.05, 95% CI: 0.98–1.13; *P* = 0.15) (Figure 6B).

### Death within 60 days

Deaths within 60 days postoperation were reported in four studies. The fixed-effect model was used to calculate pooled estimates, which included 3724 patients. No significant difference was noted between the two programs (RR = 0.83, 95% CI: 0.35–2.00; *P* = 0.68) (Figure 6C).

## DISCUSSION

The results of our meta-analysis indicated marginal significant differences in DFS between the group of FU/capecitabine-based preoperative chemoradiotherapy and the group that employed oxaliplatin (RR =0.89, 95% CI: 0.78–1.00; *P* = 0.05). As a result, we believe that the employment of OX in preoperative chemoradiotherapy for LARC likely increases DFS rates. A larger sample size is likely needed to obtain significant differences [29, 30]. Negative DFS results were obtained from ACCORD-12 [18, 25], JIAO 2015 [21] and PETACC-6 [26], whereas a positive result was obtained from CAO/ARO/AIO-04 [20, 25]. CAO/ARO/AIO-04 revealed that oxaliplatin groups showed a significant DFS benefit (RR=0.82, 95% CI: 0.68–0.97, *P* < 0.05). Some researchers believed that the reason for the DFS benefit was that CAO/ARO/AIO-04 adopted oxaliplatin-based postoperative adjuvant chemotherapy regimens [20, 25, 30]. PETACC-6, which also applied to oxaliplatin in postoperative adjuvant chemotherapy, did not achieve a similar conclusion [26, 30]. In addition to the above reason, an increased intensity and good compliance in the adjuvant chemotherapy regimens was noted for the treatment groups compared with the control group in CAO/ARO/AIO-04, which may explain the difference [20, 30].

The meta-analysis results showed that the arm that employed oxaliplatin exhibited a significantly decreased distant metastasis rate (RR=0.79, 95%CI: 0.67–0.94, *P* = 0.007). Furthermore, compared with FU alone, oxaliplatin plus FU exhibited a significantly increased pCR rate (RR = 1.24, 95% CI: 1.02–1.51; *P* = 0.03). Five of the included trials exhibited an increase in pCR rates (the other 2 trials showed equal pCR rates). However, as a surrogate index for curative effect, the metastasis rate and pCR do not completely represent the long-term survival benefit, e.g., overall survival [31]. Additionally, the incidence of grade 3–4 acute toxicity (*P* < 0.0001), especially that of diarrhea (*P* < 0.00001), was significantly increased in the oxaliplatin/FU groups. High heterogeneity regarding pCR, R0 resection rate, grade 3–4 acute toxicity and grade 3–4 diarrhea was noted in the meta-analysis primarily based on the different preoperative concurrent chemotherapy regimens and the diversity of patients in the included trials [30, 32]. No significant differences in the local recurrence rate, R0 resection rate, CRM (+), grade 3–4 radiation dermatitis, grade 3–4 hematologic, postoperative complications and death within 60 days was noted. These findings suggest that the significant increase in acute adverse reactions in oxaliplatin-based chemoradiotherapy does not cause an increase in postoperative complications. Only one trial discussed late adverse reactions [20]. Late grade 3–4 adverse reactions were reported in 21% of cases in the FU alone group and in 25% of cases in the OX/FU group, and significant differences were not noted between the two arms.

Among all previous studies, only one meta-analysis addressed this topic [28]. An et al. revealed that oxaliplatin combined with FU/capecitabine significantly increased the pCR rate (*P* = 0.04) and the incidence of late grade 3–4 adverse reactions, without a significant increase in postoperative complications. This result was consistent with our meta-analyses results. However, the previous meta-analysis only presented postoperative short-term follow-up outcomes and analyzed the pCR rate, and survival outcomes from long-term follow-up were not reported. Therefore, our study further discussed the applicability of oxaliplatin in neoadjuvant therapy for the treatment of LARC with updated comparisons of prognosis and survival between the two arms.

However, some limitations in the present meta-analyses should be noted. To date, 4 of the 7 randomized clinical trials have released DFS results (ACCORD-12, CAO/ARO/AIO-04, PETACC-6 and JIAO 2015 released DFS), 4 have released LR results (JIAO 2015, ACCORD-12, NSABP R-04, CAO/ARO/AIO-04), 3 have released DMR results (JIAO 2015, ACCORD-12, CAO/ARO/AIO-04), and 1 has released late adverse reactions. No RCT reported overall survival from long-term follow-up (median survival time greater than 5 years). Therefore, a better assessment of the efficacy and late adverse reactions of oxaliplatin in neoadjuvant therapy for LARC relies on the subsequent long-term follow-up findings of the phase III clinical trials mentioned above.

In conclusion, neoadjuvant CRT with OX improves DFS for LARC, decreases distant metastases, and significantly increases the pCR rate. However, a significant increase in toxicity was observed. Therefore, the use of oxaliplatin in neoadjuvant chemoradiotherapy for LARC is promising based on the present evidence. LARC patients are likely to benefit from treatment of CRT regimens with oxaliplatin.

## MATERIALS AND METHODS

### Inclusion and exclusion criteria

All randomized controlled trials, published or unpublished, were eligible for this meta-analysis. All trials that evaluated the efficacy and safety of FU-based CRT with or without OX as neoadjuvant treatment for LARC were included. Non-original, quasi-randomized, non-randomized, or single-arm phase II trials were excluded.

Primary outcomes included disease free survival (DFS), distant metastasis rate (DMR) and local recurrence rate (LRR), whereas secondary outcomes included pathologic complete response (pCR), R0 resection, positive circumferential resection margin (CRM+), grade 3–4 acute toxicity, grade 3–4 diarrhea, grade 3–4 radiation dermatitis, grade 3–4 hematologic toxicity, death within 60 days, and postoperative complications.

### Literature search

Our electronic search imposed no restrictions regarding language, publication status or publication year. Articles were obtained by searching publications from EMBASE, PubMed, the Cochrane Library and Web of Science published through December 30, 2015. Emtree or MeSH terms were used throughout the search schemes, which were adjusted appropriately in various electronic records. In addition to electronic searches for original papers, the references of involved studies were also reviewed to identify potentially eligible articles. Moreover, unpublished abstracts from the following major academic conferences were searched: ASCO (American Society of Clinical Oncology), ESSO (European Society of Surgical Oncology), ESTRO (European Society for Radiation Oncology), ASTRO (American Society for Radiation Oncology) and ESMO (European Society for Medical Oncology). We also contacted the first author or corresponding author to obtain information if the research results were unclear or more information was needed.

### Data extraction and assessment of the risk of bias

The data extraction was performed independently by two reviewers (Ling Cao and Yong-Jing Yang). Disagreements were resolved by a third reviewer (Zhi-Rui Zhou). The following information was extracted from each included trial: participant eligibility, study design, baseline characteristics, duration of follow-up, and the number of events for all outcomes and interventions. If the results were reported in multiple publications, we extracted data from all the publications.

The quality of included studies was independently evaluated by two investigators. Assessment of the bias of the included studies was based on the Cochrane Handbook for Systematic Reviews of Interventions [32]. When needed, a third reviewer was employed to resolve disagreements. On the basis of the assessment of general sequence allocation, allocation concealment, incomplete data addressed, outcome assessment blinding (detection bias), participants and personnel blinding (performance bias), presence of biases in reports and other bias sources that may affect the validity of the study, the studies were classified as having a high, unclear or low risk of bias.

### Statistical analysis

RevMan 5.3 software (The Cochrane Collaboration, UK) was used for statistical analysis. Risk ratios (RR) and 95% confidence intervals (CI) were calculated for count data. I-square and Chi-square tests were employed to assess studies-shared heterogeneity. If heterogeneity was not detected (*P* > 0.10, *I*^2^ < 50%), the analysis was performed using the fixed-effect model. Otherwise, a random-effect model was used. With heterogeneity, the following three potential sources were explored: methodological, clinical and statistical. If excessive heterogeneity was noted, descriptive analysis was employed for the meta-analysis.

## Supplementary Materials


