# Supplementary material for: Fluorouracil-based neoadjuvant chemoradiotherapy with or without oxaliplatin for treatment of locally advanced rectal cancer: An updated systematic review and meta-analysis
Source: Oncotarget. 2016 Jun 14;7(29):45513–24. doi: 10.18632/oncotarget.9995 (PMC5216738; doi:10.18632/oncotarget.9995)
Supplement: Supplementary file 1 [file oncotarget-07-45513-s001.pdf]

Fluorouracil-based neoadjuvant chemoradiotherapy with or without oxaliplatin for treatment of locally advanced rectal cancer: An updated systematic review and meta-analysis

Supplementary Materials

Appendix: PubMed search terms

#1 ((((((((((((((((((((Neoplasm, Rectal[Title/Abstract]) OR Rectal Neoplasm[Title/Abstract]) OR Rectum Neoplasms[Title/Abstract]) OR Neoplasm, Rectum[Title/Abstract]) OR Neoplasms, Rectum[Title/Abstract]) OR Neoplasms, Rectum[Title/Abstract]) OR Neoplasms, Rectal[Title/Abstract]) OR Rectal Tumors[Title/Abstract]) OR Rectal Tumor[Title/Abstract]) OR Tumor, Rectal[Title/Abstract]) OR Tumors, Rectal[Title/Abstract]) OR Cancer of Rectum[Title/Abstract]) OR Rectum Cancers[Title/Abstract]) OR Rectal Cancer[Title/Abstract]) OR Cancer, Rectal[Title/Abstract]) OR Cancers, Rectal[Title/Abstract]) OR Rectal Cancers[Title/Abstract]) OR Rectum Cancer[Title/Abstract]) OR Cancer, Rectum[Title/Abstract]) OR Cancers, Rectum[Title/Abstract]) OR Cancer of the Rectum[Title/Abstract])) OR “Rectal Neoplasms”[Mesh]

#2 oxaliplatin [Title/Abstract]

#3 ((((((((((((((((((((((((((Chemoradiotherapies[Title/Abstract]) OR Radiochemotherapy[Title/Abstract]) OR Radiochemotherapies[Title/Abstract]) OR Concurrent Chemoradiotherapy[Title/Abstract]) OR Chemoradiotherapies, Concurrent[Title/Abstract]) OR Chemoradiotherapy, Concurrent[Title/Abstract]) OR Concurrent Chemoradiotherapies[Title/Abstract]) OR Synchronous Chemoradiotherapy[Title/Abstract]) OR Chemoradiotherapies, Synchronous[Title/Abstract]) OR Chemoradiotherapy, Synchronous[Title/Abstract]) OR Synchronous Chemoradiotherapies[Title/Abstract]) OR Concurrent Radiochemotherapy[Title/Abstract]) OR Concurrent Radiochemotherapies[Title/Abstract]) OR Radiochemotherapies, Concurrent[Title/Abstract]) OR Radiochemotherapy, Concurrent[Title/Abstract]) OR Concomitant Chemoradiotherapy[Title/Abstract]) OR Chemoradiotherapies, Concomitant[Title/Abstract]) OR Chemoradiotherapy, Concomitant[Title/Abstract]) OR Concomitant Chemoradiotherapies[Title/Abstract]) OR Concomitant Radiochemotherapy[Title/Abstract]) OR Concomitant Radiochemotherapies[Title/Abstract]) OR Radiochemotherapies, Concomitant[Title/Abstract]) OR Radiochemotherapy, Concomitant[Title/Abstract]) OR Chemoradiotherapy[Title/Abstract])) OR “Chemoradiotherapy”[Mesh])) OR ((“Neoadjuvant Therapy”[Mesh]) OR (((((((Neoadjuvant Therapies[Title/Abstract]) OR Therapies, Neoadjuvant[Title/Abstract]) OR Therapy, Neoadjuvant[Title/Abstract]) OR Neoadjuvant Treatment[Title/Abstract]) OR Neoadjuvant Treatments[Title/Abstract]) OR Treatment, Neoadjuvant[Title/Abstract]) OR Treatments, Neoadjuvant[Title/Abstract]))

#4 ((((((Randomized controlled trial) OR controlled clinical trial) OR controlled clinical trial) OR placebo) OR randomly) OR trial) OR groups

#5 #1 AND #2 AND #3 AND #4
